# Editorial: Subcellular compartmentalization of plant antioxidants and ROS generating systems, volume II

**DOI:** 10.3389/fpls.2023.1224289

**Published:** 2023-07-18

**Authors:** José M. Palma, Marta Rodríguez-Ruiz, Christine H. Foyer, Francisco J. Corpas

**Affiliations:** ^1^ Dpt. Stress, Development and Signalling in Plants, Estación Experimental del Zaidín, Spanish National Research Council (CSIC), Granada, Spain; ^2^ School of Biosciences, College of Life and Environmental Sciences, University of Birmingham, Birmingham, United Kingdom

**Keywords:** chloroplasts, mitochondria, peroxisomes, hydrogen peroxide, signalling

Plants rapidly, efficiently, and systemically transmit signals from the site of perception to distal tissues. This triggers specific abiotic and/or biotic stress tolerance responses and engenders resilience to counteract movement limitations. Within this context, the intra/extra-cellular compartmentation of reactive oxygen species (ROS), particularly hydrogen peroxide (H_2_O_2_), plays a central role in the sensing and transmission of stress signals. The ROS wave is an emerging concept that describes an auto-propagating cell-to-cell signaling pathway in which the initiating cell activates extracellular (i.e., apoplast) superoxide production *via* respiratory burst oxidase homologs (Fichman et al., 2022). H_2_O_2_ is subsequently produced and thereafter transported in or out of cells through peroxiporins and/or other systems to activate diverse signaling mechanisms (Mittler et al., 2022). While little is known about the factors that give specificity to the systemic signal, the production of H_2_O_2_ and other signals by the different intracellular compartments has been thoroughly addressed (Ravi et al., 2023).

The maintenance of oxygen homeostasis in plants is not always achievable as some tissues and cell layers therein occupy hypoxic niches, in which oxygen signaling systems control gene expression (Considine and Foyer, 2023). Cellular and/or subcellular oxidative stress occur when ROS production is increased or ROS scavenging systems are decreased in response to environmental stimuli, either biotic or abiotic. Likewise, the transient ROS concentrations within an organelle are largely determined by the presence of antioxidants, particularly in chloroplasts, mitochondria, and peroxisomes (Foyer and Noctor, 2003; Corpas et al., 2015; Noctor and Foyer, 2016; Palma and Corpas, 2021). An improved understanding of how subcellular antioxidants in plants regulate ROS signaling and the duration thereof is required to achieve sustainable crop improvement under a scenario of climate change.

The investigation of ROS metabolism in plants at the intracellular level was initially accomplished through the combination of cell biology and biochemical techniques (Foyer and Noctor, 2003; Corpas et al., 2015). This led to a large increase in foundation knowledge, that has recently been complemented by new high throughput and other cutting-edge technologies. The application of omics (e.g., transcriptomics, proteomics, and metabolomics), and combined chromatographic and mass spectrometric approaches has greatly advanced current concepts regarding the interactions between ROS/antioxidants and other metabolic pathways, and with cell signals such as hydrogen sulfide (H_2_S), nitric oxide (NO), phytohormones, and melatonin.

This volume provides an update on the landscape of the ROS and antioxidative systems of cell organelles reported earlier (Palma and Corpas, 2021). Thus, Huang et al. described the intricate interactions between ROS, NO, H_2_S, and mitochondrial DNA (mtDNA) damage. These major cell signaling molecules play a central role in maintaining the redox balance of mitochondria and the regulation of mtDNA repair pathways. Similarly, NO and H_2_S appear to participate in the epigenetic controls through interactions with various metabolic pathways, particularly those involved in the repair of nuclear and mitochondrial DNA damage.

González-Gordo et al. applied quantitative isobaric tags for relative and absolute quantitation (iTRAQ)-based protein profiling approach to analyze the proteome of peroxisomes from sweet pepper (*Capsicum annuum* L.) fruits during the ripening process. This approach enabled the identification of 57 peroxisomal proteins, of which 49 were located in the matrix, 36 had a peroxisomal targeting signal type 1 (PTS1), 8 had a PTS type 2 (PTS2), 5 did not contain any of these peptide signals, and 8 were associated with the peroxisomal membrane. Furthermore, 19 of the identified proteins were overexpressed, and 15 were repressed during the fruit ripening process. While most of these proteins were associated with ROS and antioxidant metabolism and the β-oxidation pathway, others were “new” or “unexpected” proteins.

The plastid ROS metabolism was investigated by Koh et al.. Thus, the deregulation of chlorophyll biosynthesis, which took place in plants fed with δ-aminolevulinic acid (ALA), and in the FLUORESCENT IN BLUE LIGHT *(flu*) mutant, a strain that harbors a defect in the feedback regulation of δALA synthesis, resulted in high levels of photoactive chlorophyll intermediates in the cytosol. This situation promoted the generation of singlet oxygen (^1^O_2_) in the light, leading to RNA oxidation and the expression of ^1^O_2_-responsive genes. Conversely, the transcriptome signature resulting from 3-(3,4-dichlorophenyl)-1,1-dimethylurea (DCMU) treatment, or analysis of the CHLORINA 1 (*Ch1*) mutant under moderate light conditions, did not induce the expression of ^1^O_2_-related genes.

Uzilday et al. also analyzed the ROS metabolism in chloroplasts from the C_4_-type plant *Bienertia sinuspersici* exposed to either drought or salt stress. The chlorenchyma *B. sinuspersici* cells consist of two compartments, the peripheral compartment (PC), analogous to mesophyll tissues, and the central compartment (CC), analogous to bundle sheath tissues. It was found that chloroplasts from the PC were less damaged by salt and drought stresses than those from the CC, suggesting that the PC is more tolerant to oxidative stress, possibly because it contains mechanisms that prevent or alleviate oxidation.

Finally, Jung et al. assessed the effect of the exogenous application of reduced glutathione (GSH) to the leaves of *Brassica napus* seedlings and found that it mitigated the oxidative stress caused by cadmium (Cd). The exogenous application of GSH decreased the Cd-induced ROS accumulation and enhanced antioxidant capacity, thus reducing the oxidative load of the system. The GSH treatments improved plant redox status by increasing the capacity of the ascorbate-glutathione cycle and re-establishing the hormonal balance present in the absence of Cd. Consequently, GSH applications are potentially a practical solution for the remediation of Cd-polluted soils.

This volume presents new advances in the compartmentation of proteins and processes that contribute to cellular ROS metabolism. Although significant progress has been made to date, a more extensive toolbox of molecular and cell biology techniques is required to fully understand ROS production and scavenging, their movement in and between organelles, and the integration of these activities among different cellular compartments. The development of improved *in vivo* imaging and measurement systems for key components such as glutathione and H_2_O_2_, particularly through the application of genetically-encoded redox biosensors, will be essential to fully understand the dynamic nature of redox controls and signaling in plants and to produce improved climate-resilient agricultural crops with sustainable yields.

## Author contributions

The authors conceived, contributed to write, corrected and approved the manuscript.
